# OSU-A9 inhibits pancreatic cancer cell lines by modulating p38-JAK-STAT3 signaling

**DOI:** 10.18632/oncotarget.16450

**Published:** 2017-03-22

**Authors:** Wan-Chi Tsai, Li-Yuan Bai, Yi-Jin Chen, Po-Chen Chu, Ya-Wen Hsu, Aaron M. Sargeant, Jing-Ru Weng

**Affiliations:** ^1^ Department of Medical Laboratory Science and Biotechnology, Kaohsiung Medical University, Kaohsiung 80708, Taiwan; ^2^ Center for Infectious Disease and Cancer Research, Kaohsiung Medical University, Kaohsiung 80708, Taiwan; ^3^ College of Medicine, China Medical University, Taichung 40402, Taiwan; ^4^ Division of Hematology and Oncology, Department of Internal Medicine, China Medical University Hospital, Taichung 40447, Taiwan; ^5^ Institute of Biological Chemistry, Academia Sinica, Taipei 11574, Taiwan; ^6^ Institute of Basic Medical Sciences, College of Medicine, National Cheng Kung University, Tainan 70101, Taiwan; ^7^ Department of Hospital and Health Care Administration, Chia Nan University of Pharmacy & Science, Tainan 71745, Taiwan; ^8^ Charles River Laboratories, Safety Assessment, Spencerville, OH 45887, USA; ^9^ Department of Marine Biotechnology and Resources, National Sun Yat-sen University, Kaohsiung 80424, Taiwan

**Keywords:** OSU-A9, pancreatic cancer, p38, JAK, STAT3

## Abstract

Pancreatic cancer is an aggressive malignancy that is the fourth leading cause of death worldwide. Since there is a dire need for novel and effective therapies to improve the poor survival rates of advanced pancreatic cancer patients, we analyzed the antitumor effects of OSU-A9, an indole-3-carbinol derivative, on pancreatic cancer cell lines *in vitro* and *in vivo*. OSU-A9 exhibited a stronger antitumor effect than gemcitabine on two pancreatic cancer cell lines, including gemcitabine-resistant PANC-1 cells. OSU-A9 treatment induced apoptosis, the down-regulation of Akt phosphorylation, up-regulation of p38 phosphorylation and decreased phosphorylation of JAK and STAT3. Cell migration and invasiveness assays showed that OSU-A9 reduced cancer cell aggressiveness and inhibited BxPC-3 xenograft growth in nude mice. These results suggest that OSU-A9 modulates the p38-JAK-STAT3 signaling module, thereby inducing cytotoxicity in pancreatic cancer cells. Continued evaluation of OSU-A9 as a potential therapeutic agent for pancreatic cancer thus appears warrented.

## INTRODUCTION

Pancreatic cancer is an aggressive malignancy and the fourth leading cause of cancer-related deaths worldwide [1]. Currently the most frequently used therapeutic approach is surgery followed by adjuvant chemotherapy or chemoradiotherapy [1]. However, less than 20% of patients have operable pancreatic cancer at diagnosis and 80% of the patients with localized pancreatic cancer experience recurrence within 3 years after surgery [2]. Chemotherapy remains the preferred treatment modality for patients with locally advanced or metastatic pancreatic cancer, but the mix of poor response rate and short progression-free interval time results in a five-year survival rate of less than 5% [3]. Therefore, novel and effective therapeutic avenues are urgently needed for the pancreatic cancer patients.

Indole-3-carbinol (I3C), a phytochemical commonly found in Brassica plants, has been demonstrated to interfere with oncogenic signaling pathways that are involved in cancer cell survival, invasion, migration, aggressiveness, and cell cycle propagation [4, 5]. However, its low anti-tumor activity, limited bioavailability, complex pharmacokinetics and hepatotoxicity have restricted the clinical application of I3C [4, 5]. Therefore we generated the OSU-A9 derivative by performing structural optimization of I3C, which provided considerable therapeutic advantages over its parent compound in terms of anti-tumor efficacy and metabolic stability [6]. OSU-A9 was also demonstrated to be effective against prostate, breast, oral, liver, and acute myeloid leukemia cancer cells *in vitro* and *in vivo* [6–10]. Further, studies confirmed that OSU-A9 was safe following repeated daily administration into athymic nude mice [6–10]. In addition, it has been shown to be effective in sensitizing hepatocellular carcinoma cells to Apo2L/TRAIL treatment [11].

The aim of this study was to investigate the *in vitro* and *in vivo* efficacy of OSU-A9 against pancreatic cancer cells and deciphere the mechanisms underlying its anticancer activity and test its ability to inhibit pancreatic cancer growth in athymic nude mice with BXPC-3 xenografts.

## RESULTS

### OSU-A9 shows greater anti-proliferative activity than gemcitabine

Gemcitabine is most commonly used in first-line chemotherapy against advanced pancreatic cancer and in combination with 5-fluorouracil, it demonstrated a modest survival advantage and lower toxicity [12]. However, median overall survival (OS) following treatment with gemcitabine was less than 6 months [12]. Recent clinical trials have demonstrated that newly combined chemotherapies involving gemcitabine prolong OS in advanced pancreatic cancer [13, 14].

**Figure 1:**
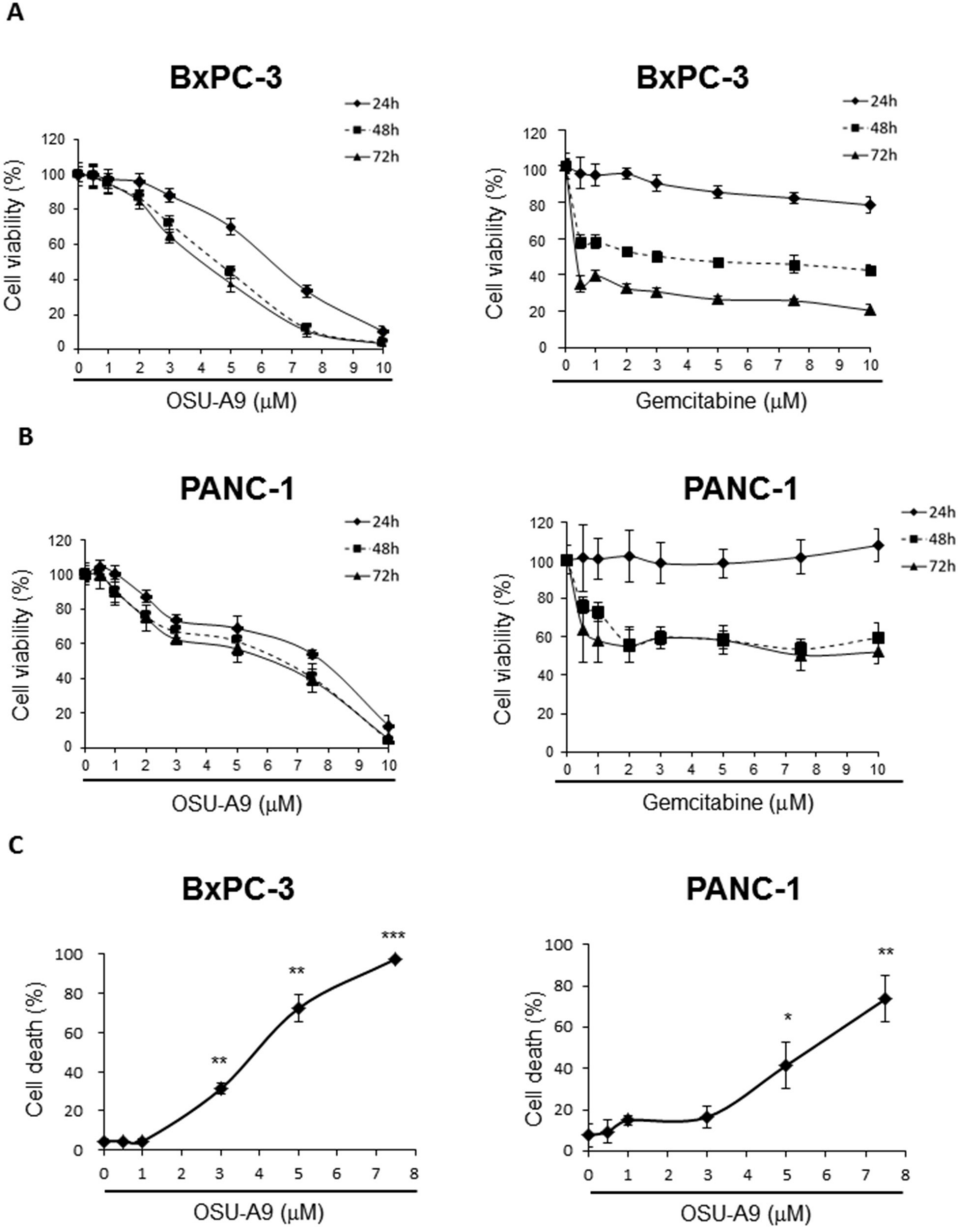
Effects of OSU-A9 on cell viability of pancreatic cancer cell lines MTT assay analyzing the effect of OSU-A9 and gemcitabine treatment on **(A)** BxPC-3 and **(B)** PANC-1 cell viability. **(C)** Trypan Blue exclusion assay to determine cell viability of BxPC-3 and PANC-1 cell lines treated with OSU-A9 for 24 h. Data are presented as mean ± S.D. from three individual experiments. * *P* < 0.05, ** *P* < 0.01, *** *P* < 0.001 compared to the control group.

Therefore, we used gemcitabine-responsive BxPC-3 and resistant PANC-1 cell lines [15] as our testing model to examine the cell viability by the MTT assays. Upon OSU-A9 treatment, we observed significant decreases in mean cell viability above 2 μM for both BxPC-3 and PANC-1 cell lines with IC_50_ values at 24 h post-treatment being 6.4 and 7.7 μM for BxPC-3 and PANC-1, respectively (Figure 1A and 1B). Nevertheless, IC_50_ values for gemcitabine exceeded those for the highest dosages of either OSU-A9 or gemcitabine that we used in these two cell lines (Figure 1A and 1B). For gemcitabine-sensitive BxPC-3 cells, treatment with OSU-A9 for 24 h exerted a stronger inhibitory effect on cell viability compared to treatment with gemcitabine, whereas, at the later time point, the inhibitory effect of OSU-A9 was less than gemcitabine. We also observed that the anti-proliferative effects of OSU-A9 were statistically significant based on the overall mean cell viability across multiple doses for 3 days in both the BxPC-3 (p < 0.0001) and PANC-1 (p < 0.0001) cell lines (Supplementry Tables 1 and 2). Trypan blue exclusion assays indicated that OSU-A9 induced BxPC-3 and PANC-1 cell deaths in a dose-dependent manner (Figure 1C). Further, we examined the combined effect of OSU-A9 and gemcitabine on the BxPC-3 and PANC-1 cell lines by calculating a combination index (CI) using the Chou-Talalay method [16] with a fixed dose ratio. We observed OSU-A9-gemcitabine synergy in the BxPC-3 cells but not in PANC-1 cells (Supplementry Figure 1).

### OSU-A9 induces apoptosis in BxPC-3 and PANC-1 cells

**Figure 2:**
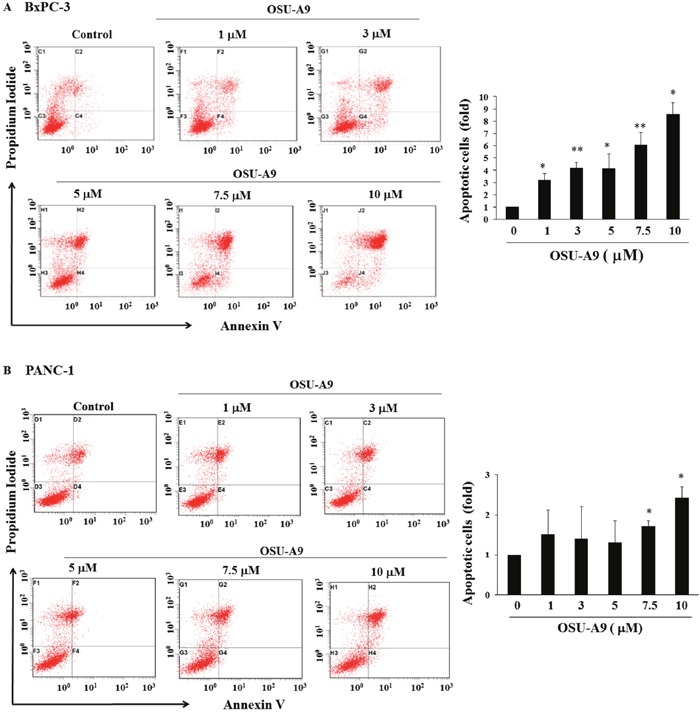
OSU-A9 induces apoptosis in BxPC-3 and PANC-1 cell lines Histograms showing quantitation of cell apoptosis in (**A**, top left panel) BxPC-3 and (**B**, bottom left panel) PANC-1 cell lines when treated with different doses of OSU-A9. Cells were treated with OSU-A9 at the indicated concentrations for 24 h, followed by Annexin V/PI staining and FACS analysis. Fold increase in apoptosis due to increasing doses of OSU-A9 treatment of BxPC-3 (**A**, top right panel) and PANC-1 cell lines (**B**, bottom right panel) in comparison to control (control value = 1) are represented by the bar graph. Data represent mean ± SEM from six independent experiments. * *P* < 0.05, ** *P* < 0.01 compared to the control group.

Flow cytometric analysis of Annexin V/propidium iodide (PI) stained OSU-A9-treated cells for 24 h indicated that OSU-A9 increased the percentage of apoptotic (Annexin V-positive) cells in a dose-dependent manner (Figure 2). This showed that the reduced cell viability by OSU-A9 treatment was due to apoptosis.

### OSU-A9 modulates PI3K/Akt and MAPK signaling pathways in the pancreatic cancer cell lines

**Figure 3:**
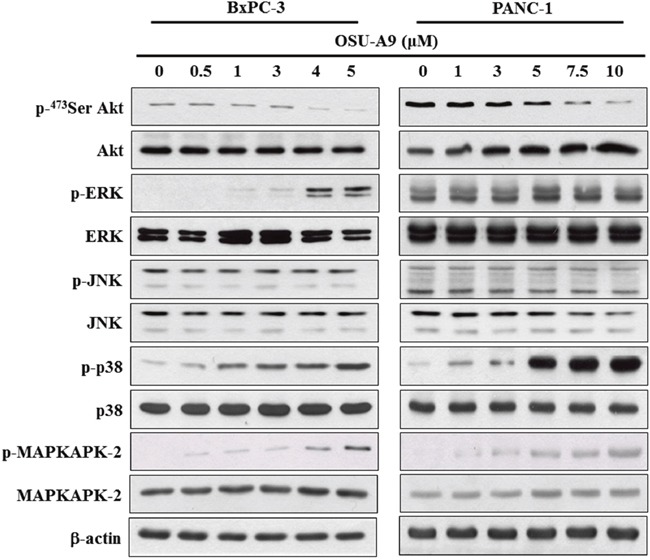
Effects of OSU-A9 on Akt and MAPK signaling pathways in pancreatic cancer cell lines Phosphorylation and expression status of Akt and MAPK signaling module (ERK, JNK, p38 and MAPKAPK2) were analyzed in BxPC-3 (left panel) and PANC-1 (right panel) cells that were treated with OSU-A9 (0-5 μM) in 5% FBS-supplemented RPMI1640 medium for 24 h. Cell lysates were immunoblotted as described in Material and Methods.

PI3K/Akt and mitogen-activated protein kinase (MAPK) signaling are central to pancreatic cancer malignancy [17, 18]. OSU-A9 has been shown to target Akt in various cancer cell lines [6, 8]. As shown in Figure 3, we observed that OSU-A9 treatment down-regulated Akt phosphorylation and up-regulated p38 and the downstream MAPKAPK-2 phosphorylation in both the BxPC-3 and PANC-1 cell lines. Western blot results indicated that whereas OSU-A9 increased phosphorylated ERK in BxPC-3, it did not affect ERK phosphorylation in PANC-1. JNK signaling was not affected in either cell line.

### p38 involves in OSU-A9-triggered JAK-STAT3 inactivation and the related consequence

**Figure 4:**
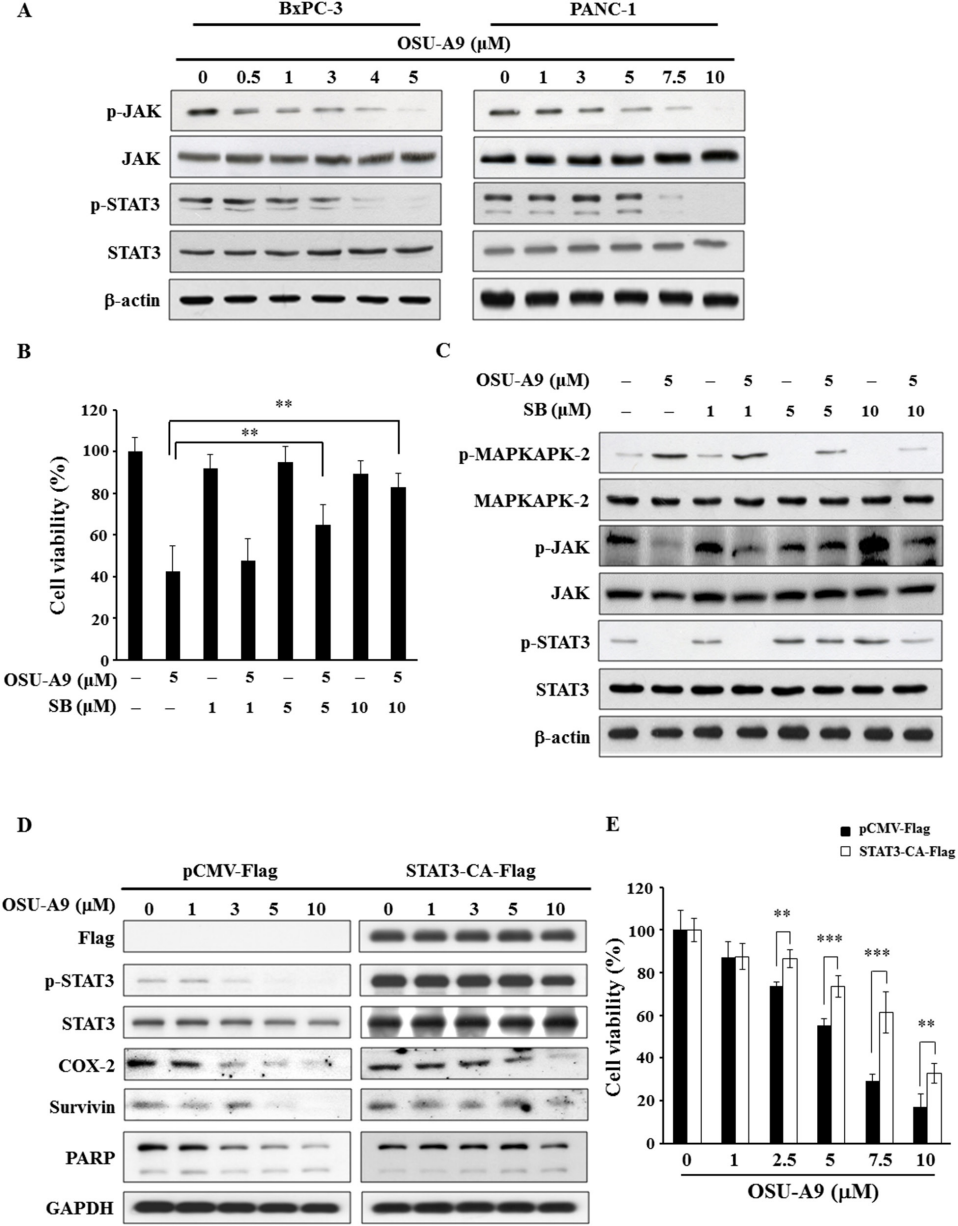
OSU-A9 down-regulates JAK-STAT3 signaling **(A)** Dose-dependent effects of OSU-A9 on JAK and STAT3 phosphorylation and expression in BxPC-3 and PANC-1 cells. **(B)** p38 inhibitor SB203580 (SB) restores cell growth inhibited by OSU-A9 in BxPC-3 cells. Cells were treated with DMSO or OSU-A9 (5 μM) in the presence of 1, 5 or 10 μM SB for 24 h and cell viability was determined by MTT assays. Data are presented as mean ± S.D. from three individual experiments. ** *P* < 0.01. **(C)** Effects of SB203580 (SB) on the phosphorylation and expression of MAPKAPK-2, JAK and STAT3 in BxPC-3 cells. Cells were treated with DMSO or OSU-A9 (5 μM) in the presence of 1, 5 or 10 μM SB for 24 h. **(D)** Western blot analysis showing phosphorylation and expression of PARP, COX-2, survivin, and STAT3 in PANC-1 cells transfected with p-CMV-Flag or STAT3-CA-Flag and treated with 0-10 μM OSU-A9 for 24 h. **(E)** STAT3-CA partially protects against OSU-A9-mediated cytotoxicity in PANC-1 cells. Percent cell viability of PANC-1 cells expressing p-CMV-Flag or STAT3-CA-Flag treated with 0-10 μM OSU-A9 are plotted. Data are presented as mean ± S.D. from three individual experiments. ** *P* < 0.01, *** *P* < 0.001.

The activation of the JAK-STAT pathway that is downstream of the Akt and MAPK signaling modules stimulates cell proliferation, potentiates malignant transformation and inhibits apoptosis in pancreatic cancer cells [19–21]. When we tested the status of JAK-STAT pathway upon OSU-A9 treatment, we observed dose-dependent decrease in JAK and STAT3 phosphorylation (Figure 4A). To further investigate the interplay between p38 and JAK-STAT3 signaling in OSU-A9-mediated cytotoxicity, we used SB203580 (a p38 inhibitor) for rescue experiments. We found that SB203580 pre-treatment of BxPC-3 cells reduced OSU-A9-induced cytotoxicity in a dose-dependent manner (Figure 4B). Further, the rescue of phosphorylated STAT3 upon SB203580 pre-treatment suggested that p38 MAPK was upstream as shown in Figure 4C. In addition, we observed that OSU-A9 treatment resulted in enhanced production of reactive oxygen species (ROS) in BxPC-3 cells based on FACS analysis and this could be suppressed by a p38 inhibitor (Supplementry Figure 2A). However, the depletion of ROS by *N*-acetylcysteine (NAC, an ROS scavenger) did not alter p38 activation by OSU-A9 (Supplementry Figure 2B). Therefore, these data suggested that OSU-A9-induced activation of p38 resulted in enhanced ROS production. Further, we analyzed mitochondrial membrane potential by staining BxPC-3 cells treated for 24h with OSU-A9 alone or in combination with NAC with a JC-1 fluorescent dye. We observed that NAC partially restored the dissipated mitochondrial membrane potential in BxPC-3 cells upon OSU-A9 treatment (Supplementry Figure 2C).

To confirm the role of STAT3 in OSU-A9-triggered cell death, we overexpressed the constitutively active form of STAT3 and found that it's downstream targets (COX-2, survivin) were significantly restored (Figure 4D). Consistent with the overall cell viability data in Figure 4E, the OSU-A9-induced PARP cleavage was mitigated (Figure 4D). These data highlighted the importance of STAT3 in OSU-A9-mediated cytotoxicity.

### OSU-A9 reduces pancreatic cancer cell aggressiveness

**Figure 5:**
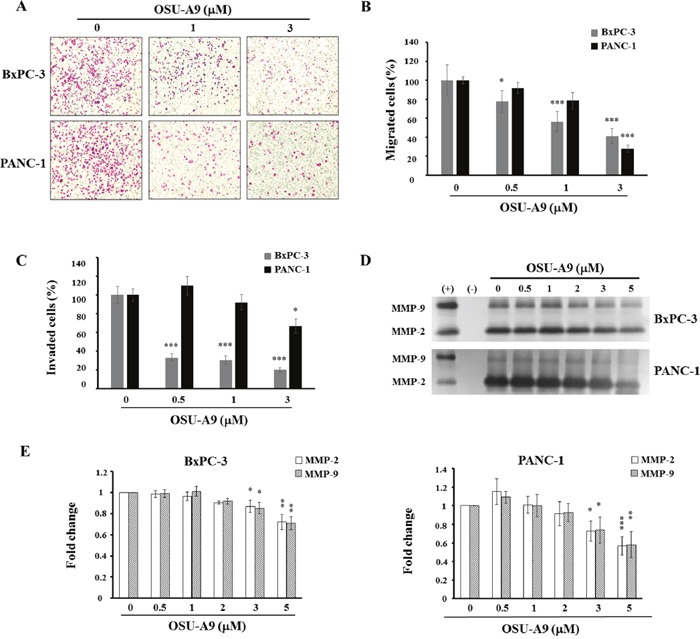
OSU-A9 mitigates aggressiveness of BxPC-3 and PANC-1 cells **(A)** BxPC-3 and PANC-1 cell migration images following treatment with 0-3 μM OSU-A9 for 24 h. Plots showing percentages of migrating **(B)** and invasive **(C)** BxPC-3 and PANC-1 cells treated with 0-3 μM OSU-A9 for 24 h. Data are presented as mean ± S.E.M. from three individual experiments. *P < 0.05, *** P < 0.001 compared to the control group. **(D)** Gelatin zymography analysis showing secreted MMP-2 and MMP-9 activity for both BxPC-3 and PANC-1 cell lines treated with 0-5 μM OSU-A9 for 24 h in conditioned media. (+) and (−) are the positive and negative control for MMP-2 and MMP-9 activity, respectively. **(E)** Bar diagrams represent fold changes in MMP-2 and MMP-9 (control value = 1) in BxPC-3 and PANC-1cell lines treated with 0-5 μM OSU-A9 for 24 h in conditioned media. Data are presented as mean ± S.E.M. from three individual experiments. *P < 0.05, ** *P* < 0.01, *** *P* < 0.001 compared to the control group.

Previous studies have shown that p38 activation enhances PP2Acα up-regulation resulting in MMP mRNA decay [22] and the inhibition of JAK/STAT3 that are both important for pancreatic cancer progression [23]. We therefore used migration and invasion assays to examine the effects of OSU-A9 on cell aggressiveness and found that it (a) significantly inhibited BxPC-3 and PANC-1 cell migration in a dose-dependent manner compared to the controls (Figure 5A and 5B) and (b) inhibited overall cell invasion (Figure 5C) at a dose that neither affected viability nor induced apoptosis.

Since matrix metalloproteinases (MMP) are linked with cancer cell migration and invasion [24], we performed gelatin zymography to analyze the levels of secreted MMP-2 and MMP-9 following OSU-A9 treatment of BxPC-3 and PANC-1 cells for 24 h and found reduced MMP-2 and MMP-9 proteolytic activity (Figure 5D). Quantitative data for each cell line are shown in Figure 5E.

### OSU-A9 inhibits BxPC-3 xenograft growth in nude mice

**Figure 6:**
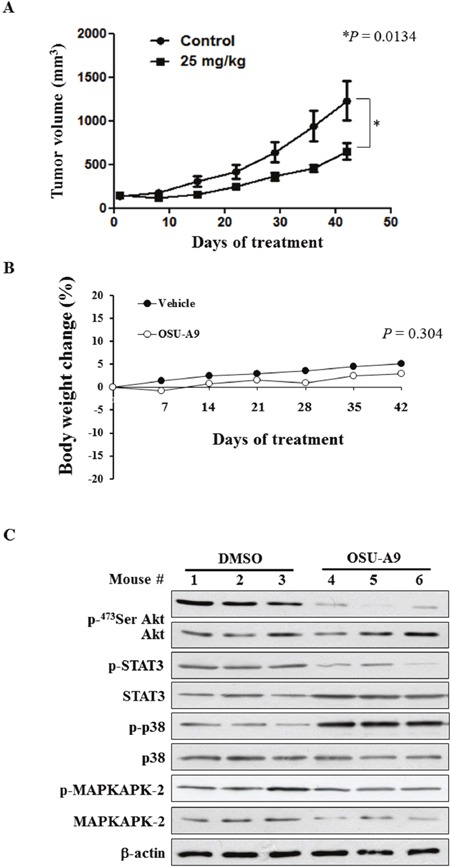
*In vivo* efficacy of OSU-A9 in BxPC-3 xenografted mice **(A)** Tumor volume data plotted for mice with BxPC-3 xenografts treated with either DMSO or OSU-A9 (25 mg/kg) per day via oral gavage analyzed at different time points from 0-42 days. **(B)** Plot showing change in body weight of xenografted mice in the DMSO- and OSU-A9-treated groups. **(C)** Western blot showing phosphorylation status and expression of Akt, STAT3, p38, and MAPKAPK-2 proteins as analyzed from tumor protein extracts of 3 mice in each treatment group.

To investigate the *in vivo* effects of OSU-A9, BxPC-3 cells were xenografted into 20 male athymic nude mice separated into two groups of 10 animals each. We observed that mice treated with an oral gavage of OSU-A9 (25 mg/kg), once daily had smaller tumor volumes compared to control group mice at the study endpoint (*P* = 0.0134) (Figure 6A). Although the mice in the OSU-A9 group had lower body weight gain compared to control group mice over the duration of the experiment, the terminal body weight between the two groups was statistically insignificant (*P* = 0.304) (Figure 6B). At the end point of the experiment, the tumors as wells as vital organs including liver, heart, kidney, and lung from all mice were examined macroscopically and blood tests were performed to evaluate serum chemistry and hematological parameters. Our analysis demonstrated no gross changes or differences in clinical pathology values (serum chemistry and hematology results presented in Supplementry Table 3) due to OSU-A9 treatment. To examine the biological markers *in vivo*, we randomly selected tumor tissues from 3 mice in each group and examined the signaling pathways. Treatment with OSU-A9 resulted in the downregulation of Akt and STAT3 phosphorylation and the upregulation of p38 and MAPKAPK-2 phosphorylation (Figure 6C), confirming the *in vitro* results.

## DISCUSSION

STATs belong to a family of cytoplasmic proteins with SH2 (Src Homology-2) domains that act as signal messengers and transcriptional factors involved in cellular responses to extracellular cytokines. The STAT3 gene plays an important role in mediating cellular processes like cell growth, apoptosis, and differentiation [25]. The oncogenic STAT3 protein is constitutively activated in many human cancers, including pancreatic cancer [26, 27]. Recent studies have demonstrated that the constitutive activation of STAT3 and the subsequent promotion of tumor cell growth, invasion, and metastasis in pancreatic cancer patients results in high mortality [28, 29]. The JAK–STAT pathway is a ubiquitous, evolutionarily conserved signaling cascade. JAKs are receptor-associated tyrosine kinases that phosphorylate each other and the receptor [30].

The latent transcription factor STAT3 is recruited to the phosphorylated receptor and transphosphorylated by JAK [31]. JAK-STAT3 pathway activation in patients with malignancies predisposes worst outcomes. For example, activated JAK-STAT3 signaling is associated with reduced overall survival in pancreatic cancer patients [19]. In our study, OSU-A9 inactivated both JAK and STAT3 in BxPC-3 and PANC-1 cells (Figure 4A). The inhibition of STAT3 phosphorylation by OSU-A9 could be restored by ectopic overexpression of constitutively active JAK (Supplementry Figure 3) demonstrated that the JAK-STAT3 axis was suppressed by OSU-A9. However, in addition to JAK, STAT3 can also be activated by other kinases including mammalian target of rapamycin (mTOR), p38, and MEK [18, 19]. The p38 MAP kinase has divergent regulatory roles on STAT3 activation. Gollob and colleagues have reported that the p38 MAP kinase inhibitor eliminates both STAT1 and STAT3 serine phosphorylation in response to IL-12 and IL-2 [32]. Further, p38 MAP kinase plays a critical role in regulating STAT serine phosphorylation in the lymphocytes [32]. Conversely, p38 has also been shown to be a negative regulator for STAT3 signaling [33]. In the context of SOCS3, IL-6, or IL-10 modulation [34], the inhibitory effect of LPS and tumor necrosis factor α (TNFα) on IL-6-induced STAT3 activation is rescued by p38 inhibition [35, 36]. Similar to previously reported finding [22], we found that p38 inhibition by SB203580 decreased the OSU-A9-mediated JAK and STAT3 dephosphorylation (Figure 4C), suggesting that p38 was upstream and had negative regulatory effect on STAT3 activation. Interestingly, we also observed an increase in the basal level of STAT3 serine phosphorylation in the presence of SB203580 alone. This could be due to increased MKK6 activity that is enhanced by SB203580 [37]. Since MKK6 is a major upstream activator of p38, the increased level of STAT3 phosphorylation induced by SB203580 may be due to the MKK6-induced activation of an unidentified serine/threonine kinase that is distinct from p38 and not inhibited by SB203580 [32]. Taken together, these findings implicate that activated p38 by OSU-A9 contributed to inactivation of JAK-STAT3.

Regarding the induction of ROS and p38 activation in the present study, it is interesting to note that co-treatment with an ROS scavenger (NAC) did not alter p38 activation (Supplementry Figure 2B). This observation implies OSU-A9-induced ROS production was a consequce, rather than a cause, of p38 activation in pancreatic cancer cells. However, the mechanism by which OSU-A9 activates p38 remains undefined.

During metastasis, cancer cells go through a process of detachment, migration, invasion and adhesion that is affected by multiple biochemical events and parameters. In pancreatic cancer, down-regulation of STAT3 expression suppressed the growth and invasiveness of cancer cells, both *in vitro* and *in vivo* [27, 38]. In the present study, we observed that OSU-A9 significantly inhibited cell migration and invasiveness of BxPC-3 and PANC-1 cells (Figure 5). The impaired cell migration and tumor invasion could partly be attributed to STAT3 inactivation by OSU-A9.

The MMPs consist of at least 26 proteases divided into four groups: collagenases, gelatinases, stromelysins and matrilysins that play key roles in tumor metastasis [39]. MMP-2 expression is upregulated by a constitutively activated STAT3 that binds directly to the MMP-2 gene promoter [40]. STAT3 activation also regulates MMP-9 and MMP-1 expression [41, 42]. These data highlight the central role of STAT3 in actively promoting cellular invasion. Further, MMP-7 (matrilysin) triggers the invasive and metastatic capacity of pancreatic ductal adenocarcinoma cells via a STAT3-dependent mechanism that is also demonstrated in the prostate, breast and pancreatic cancer cell lines [43–45]. The gelatin zymography data (Figure 5D) suggest that inhibition of MMP-2 (gelatinase A) and MMP-9 (gelatinase B) may be responsible for the repression of BxPC-3 invasiveness.

Although the role of STAT3 in pancreatic cancer aggressiveness [46–48] and the effectiveness of STAT3 signal-inhibiting agents [49, 50] have previously been demonstrated, this is the first study to our knowledge to address the role and the potential therapeutic application of the p38-JAK-STAT3 pathway in pancreatic cancer therapy. Though gemcitabine is known to induce apoptosis via p38 activation in pancreatic cancer cells, STAT3 phosphorylation status is not altered [51, 52]. Recently, solamargine, a steroidal alkaloid glycoside extracted from the Chinese medicinal herb *Solanum nigrum* L. was shown to induce p38 phosphorylation and STAT3 dephosphorylation in lung cancer cells that can be blocked by SB203580 [53]. However, changes in JAK or phosphorylated JAK were not assessed in that study.

For gemcitabine-sensitive BxPC-3 cells, treatment with OSU-A9 for 24h exerted a stronger inhibitory effect on cell viability compared to gemcitabine (IC_50_ values of 6.4 versus > 35 μM). However, at a later time point, the inhibitory effects of OSU-A9 were milder than gemcitabine on the BxPC-3 cells. The delayed effect of gemcitabine on BxPC-3 cells could reflect its mode of action as a nucleoside analog. In contrast, p38-JAK-STAT3 inhibition by OSU-A9 likely explains its immediate cytotoxic activity compared to gemcitabine. For gemcitabine-resistant PANC-1 cells, OSU-A9 induced a dose-dependent inhibitory effect on cell viability. As expected, gemcitabine had no effect on PANC-1 viability.

Drug combinations are widely used in the treatment of refractory tumors, including those associated with pancreatic cancer [54]. Therefore, when we combined OSU-A9 with gemcitabine to determine potential synergistic effects, OSU-A9-gemcitabine showed synergistic effects in BxPC-3 cells but not in PANC-1 cells (Supplementry Figure 1). In PANC-1 cells, the cytotoxic effect must have been primarily due to OSU-A9. PANC-1 cells have activating point mutations at codon 12 of the k-*ras* gene, but BxPC-3 cells do not have a *ras* mutation [55]. Mutations in the k-*ras* gene are predictive biomarkers for poor clinical outcomes in pancreatic cancer patients treated with gemcitabine-based chemotherapy [56]. Whether the discriminatory synergistic effects of OSU-A9-gemcitabine treatment are linked to k-*ras* mutations needs further study.

In summary, we found that OSU-A9 exhibited a stronger anti-proliferative effect than gemcitabine on pancreatic cancer cells. Treatment with OSU-A9 induced apoptosis, the down-regulation of Akt phosphorylation, the up-regulation of p38 phosphorylation, the inhibition of JAK and STAT3 phosphorylation, a reduction in pancreatic cancer cell aggressiveness, and the blocking of BxPC-3 xenograft growth in nude mice. In conclusion, our study suggests that the negatively-regulated JAK-STAT3 axis by p38 plays a significant role in OSU-A9-mediated cytotoxicity in pancreatic cancer and supports future efforts to test therapeutic applications of OSU-A9 in pancreatic cancer patients.

## MATERIALS AND METHODS

### Cell culture

BxPC-3 and PANC-1 human pancreatic cells were purchased from the American Type Culture Collection (Manassas, VA) and cultured in RPMI-1640 supplemented with 10% fetal bovine serum (FBS) (Gibco, Grand Island, NY), 1% sodium pyruvate (SP), and 1% penicillin/streptomycin (P/S) in 5% CO_2_ at 37°C.

### Reagents

OSU-A9 was provided by one of the co-authors, Dr. J.-R. Weng [9]. Identity and purity (≥99%) was verified by proton nuclear magnetic resonance, high-resolution mass spectrometry and elemental analysis. Primary antibodies for Akt, p-Akt (Ser473), ERK, p-ERK (Thr202/Tyr204), PARP, JNK, p-JNK (Thr183/Tyr185), p38 MAPK, p-p38 MAPK (Thr180/Tyr182), Caspase-3, MAPKAPK-2, p-MAPKAPK-2 (Thr334), JAK, p-JAK (Tyr1007/Tyr1008), STAT3, p-STAT3 (Ser727) were from Cell Signaling Technologies (Beverly, MA) whereas β-actin was from Sigma-Aldrich (St. Louis, MO). The p38 mitogen-activated protein kinase inhibitor SB203580 was purchased from Sigma-Aldrich. pCMV-Flag and STAT3-CA-Flag were purchased from Addgene (Cambridge, MA). The JAK2^V617F^-MSCV-IRES-GFP vector was kindly provided by Dr. Yen, Jeffrey J.Y. (Academia Sinica, Taiwan).

### MTT assays

For cell growth analysis, cells were seeded in 96-well flat-bottom plates (5×10^3^/well) and treated with various concentrations of test agents for the indicated time intervals. To quantify cell viability, old medium was replaced with 150 μL fresh medium containing 10% MTT solution (Sigma-Aldrich) [9]. After 1 h incubation at 37°C, the MTT solution was removed and the intracellular formazan crystals were solubilized with 100 μL DMSO. The absorbance levels for each sample at 595 nm were measured using an enzyme-linked immunosorbent assay reader (Power Wave 340; Bio-Tek Instruments, Inc.) and microplate reader (Bio-Rad Laboratories, Richmond, CA). The data were obtained from six replicates.

### Trypan blue exclusion assays

After trypsinization to detach adhering cells, all the cells (floating and attached) were pelleted and resuspended in culture medium loaded with 0.2% trypan blue. Percentages of dead cells were determined by counting total and blue-stained cells with a hemocytometer.

### AnnexinV/PI assays

Drug-treated cells were stained with AnnexinV and propidium iodide (1 μg/mL) [6] and analyzed by FACS using a BD FACSAria flow cytometer and ModFitLT V3.0 software (Becton Dickinson, Germany).

### Immunoblotting

Western blot analyses were performed as previously described [10]. Briefly, a total of 1×10^6^ cells were treated with OSU-A9 or DMSO (control) for 24 h. Cells were washed twice with ice-cold phosphate-buffered saline, harvested, and disrupted in RIPA lysis buffer containing 1% protease inhibitors. Cell lysates were centrifuged at 12000 rpm for 30 min at 4°C. Protein concentrations were measured by Bio-Rad protein assays. Equal amounts of protein samples were separated by sodium dodecyl sulfate−polyacrylamide gel electrophoresis (SDS-PAGE) and electrotransferred onto nitrocellulose (NC) paper. After 1 h blocking with TBST (TBS containing 0.1% Tween 20) containing 5% nonfat milk, membranes were incubated with corresponding primary antibodies overnight, washed thrice with TBST buffer and further incubated with secondary antibodies. Signals were visualized with enhanced chemiluminescence substrate (PerkinElmer, Boston, MA, USA).

### Transient transfection

PANC-1 cells (2 × 10^5^/3 mL) were transfected with indicated plasmid using Lipofectamine 2000 (Invitrogen) according to the manufacturer's protocol. Cells were incubated for 24 h and then treated with OSU-A9 for another 24 h.

### Invasion and migration assays

Cancer cell invasiveness was determined using Matrigel invasion chambers (BD Bio, Germany) according to the manufacturer's instructions. Briefly, Matrigel (1 mg/ml) was added into transwell inserts and incubated overnight at 37°C for consolidation. Cells (2×10^4^) mixed with serum-free medium containing OSU-A9 or DMSO were plated in the upper chamber and 500 μl RPMI-1640 medium containing 10% FBS was added to the bottom chamber. After 24 h incubation at 37°C, non-invasive cells were removed from the upper chamber with a cotton swab. Invasive cells that adhered to the inserted membranes were fixed and stained with 1% formaldehyde, 1% methanol and 0.05% crystal violet made in PBS solution for 30 minutes. Degree of invasiveness were quantified by counting cell numbers in five random fields. All experiments were repeated thrice. The migration assay procedure was similar to that described for the invasion assays except that Matrigel was not added to the transwells and that the total incubation time was 4 h.

### Zymography

Gelatin zymography was used to examine gelatinolytic activity in the culture medium [57]. After treating the cell lines with OSU-A9 for 24h, conditioned medium was subjected to SDS-PAGE analysis using 0.2% gelatin-containing gels. Samples were incubated for 30 min at 37°C in SDS sample buffer without a reducing agent and then electrophoresed on 10% polyacrylamide gels at 4°C. Following electrophoresis, gels were washed in 2.5% Triton X-100 to remove the SDS, incubated for 22 h at 37°C in 50 mM Tris-HCl (pH 7.5) containing 0.15 M NaCl, 10 mM CaCl_2_ and 0.02% NaN_3_ followed by staining with 0.1% Coomassie brilliant blue R250. Pre-stained molecular weight standards (Bio-Rad) were included in each gel. MMP-9 and MMP-2 gelatinolytic activity was visualized as clear bands at molecular weights of 92-100 kDa for MMP-9 and 62-72 kDa for MMP-2. Bands were quantitated by scanning wet gels with a densitometer. The conditioned medium from the highly invasive breast cancer cell line (MDA-MB-231) was used as positive control and the serum-free medium containing DMSO alone as negative control.

### *In vivo* study

Male NCr athymic nude mice (5-7 weeks old) were obtained from the U.S. National Cancer Institute (Frederick, MD). Mice were housed in an alternating 12 h light/dark cycle with ad libitum access to sterile food and water. All experimental procedures were performed in accordance with protocols approved by the Institutional Laboratory Animal Care and Use Committee of The Ohio State University. Individual mice under isoflurane anesthesia were inoculated subcutaneously in the right flank with 1 × 10^6^ BxPC-3 cells in 0.1 mL serum-free medium containing 50% Matrigel. As tumors became established (mean starting tumor volume, 109 ± 10 mm^3^), mice were randomly assigned to OSU-A9 treatment (n = 10) or DMSO control (n = 10) groups. Mice received daily gavage treatments with either OSU-A9 (25 mg/kg body weight) or DMSO (1% in soybean oil). Tumors were measured once per week using calipers. Volumes were calculated using a standard formula: width^2^ × length × 0.52. Body weights were measured weekly. All mice were euthanized when the tumor volume of any one mouse reached the equivalent of 10% body weight. At the time of the terminal sacrifice, blood samples from each mouse were submitted to The Ohio State University Veterinary Clinical Laboratory Services for serum chemistry and hematological parameter analysis. Macroscopic evaluation of the tumors and vital organs including liver, heart, kidney and lung and interpretation of clinical pathology endpoints were performed by a board-certified veterinary pathologist. Portions of each tumor were snap-frozen in liquid nitrogen and stored at −80°C until needed for Western blot analysis of relevant biomarkers.

### Statistical analysis

Factorial repeated-measures ANOVAs was applied to test if there was an overall significant difference between the means of cell viability at different doses (0, 0.5, 1, 2, 3, 5, 7.5, 10 μM). When significant variation between dose and day was indicated by ANOVA, post hoc t-tests adjusted with a Bonferroni correction for multiple pairwise comparisons were performed to test the differences between groups. For *in vivo* study, differences among group means of tumor volumes and weight changes were analyzed using two-way ANOVAs followed by Tabular tests. All tests were 2-tailed and significance was defined as p < 0.05. Statistical analyses were performed using SPSS version 18 and Excel 2010.

## SUPPLEMENTARY MATERIALS FIGURES AND TABLES


