# Supplementary material for: OSU-A9 inhibits pancreatic cancer cell lines by modulating p38-JAK-STAT3 signaling
Source: Oncotarget. 2017 Mar 22;8(17):29233–46. doi: 10.18632/oncotarget.16450 (PMC5438726; doi:10.18632/oncotarget.16450)
Supplement: Supplementary file 1 [file oncotarget-08-29233-s001.pdf]

OSU-A9 inhibits pancreatic cancer cell lines by modulating p38-JAK-STAT3 signaling

SUPPLEMENTARY DATA

Analysis of combined drug effect

Drug synergy was determined with isobolograms, based on the median-effect principle described by Chou and Talalay [16]. Effects of individual or combined treatment with OSU-A9 and/or gemcitabine on BxPC-3 and PANC-1 cells were determined by MTT assays and analyzed with isobolograms using CalcuSyn software (v 1.0) (Biosoft, Ferguson, MO). CalcuSyn analyzed the dose effect of OSU-A9 (1.25, 2.5, 5, 10 µM) and gemcitabine (12.5, 25, 50, 100 µM) in PANC-1 cells; OSU-A9 (0.625, 1.25, 2.5, 5 µM) and gemcitabine (2.5, 5, 10, 20 µM) in BxPC-3 cells. Dose effects of OSU-A9 and gemcitabine with fixed dose ratios were calculated using a combination index (CI) that identified dual-drug synergism, antagonism, or additivity.

Mitochondrial membrane potential (ΔΨm) and reactive oxygen species (ROS) generation

For mitochondrial membrane potential analysis, cells ($2x10^5$/3 mL) were treated with DMSO or OSU-A9 with or without 10 µM SB203580 (SB) for 3 h and washed twice with PBS, followed by staining with JC-1 dye (2.5 µM) for 15 mins in darkness at 37°C. For ROS determinations, cells ($2x10^5$/3 mL) were treated with DMSO or OSU-A9 with or without 10 mM *N*-acetylcysteine (NAC) for 3 h and washed twice with PBS, followed by staining with DCFH-DA (5 µM). Cells in both analyses were assessed for fluorescence intensity using a flow cytometer (Becton Dickinson, Germany).

Transient transfection for JAK overexpression

PANC-1 cells (2 x 10^5/3 mL) were transfected with Lipofectamine 2000 (Invitrogen) according to the manufacture's protocol and then cultured in a six-well plate for 24 h.

Immunoblotting

Cells ($1.5x10^5$/mL) were treated with DMSO or OSU-A9 with or without 10 mM *N*-acetylcysteine (NAC) for 3 h and washed twice with PBS. The total cell lysates were collected and applied for the further detection.

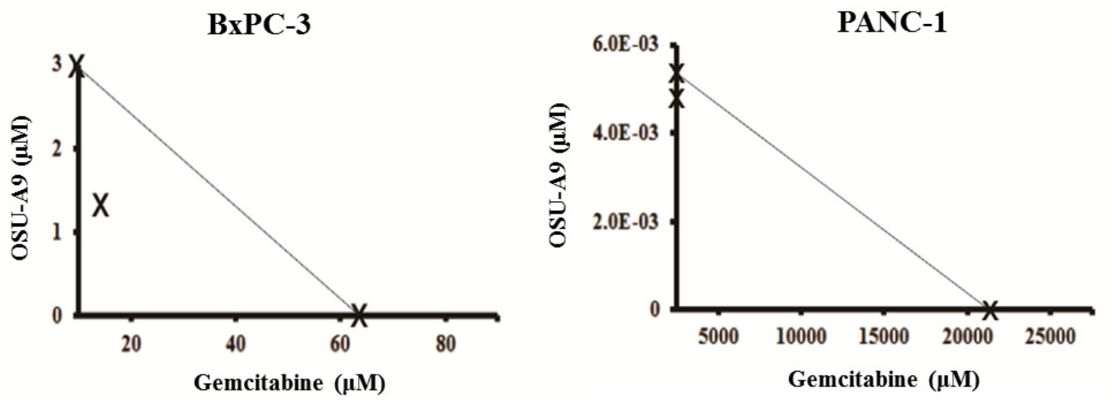

Supplementary Figure 1: Isobolograms showing the combination of OSU-A9 and gemcitabine for both BxPC-3 and PANC-1 cells. Cells were treated with OSU-A9 alone, gemcitabine alone, or OSU-A9 and gemcitabine in combination for 24 h. Cell viability was assessed by MTT assays. Straight lines indicate individual OSU-A9 and gemcitabine dosages required to achieve 90% growth inhibition. Combination index (CI) values are shown by points above (indicating antagonism) or below (indicating synergy) that line. X symbols indicate effect points (ED_{50}).

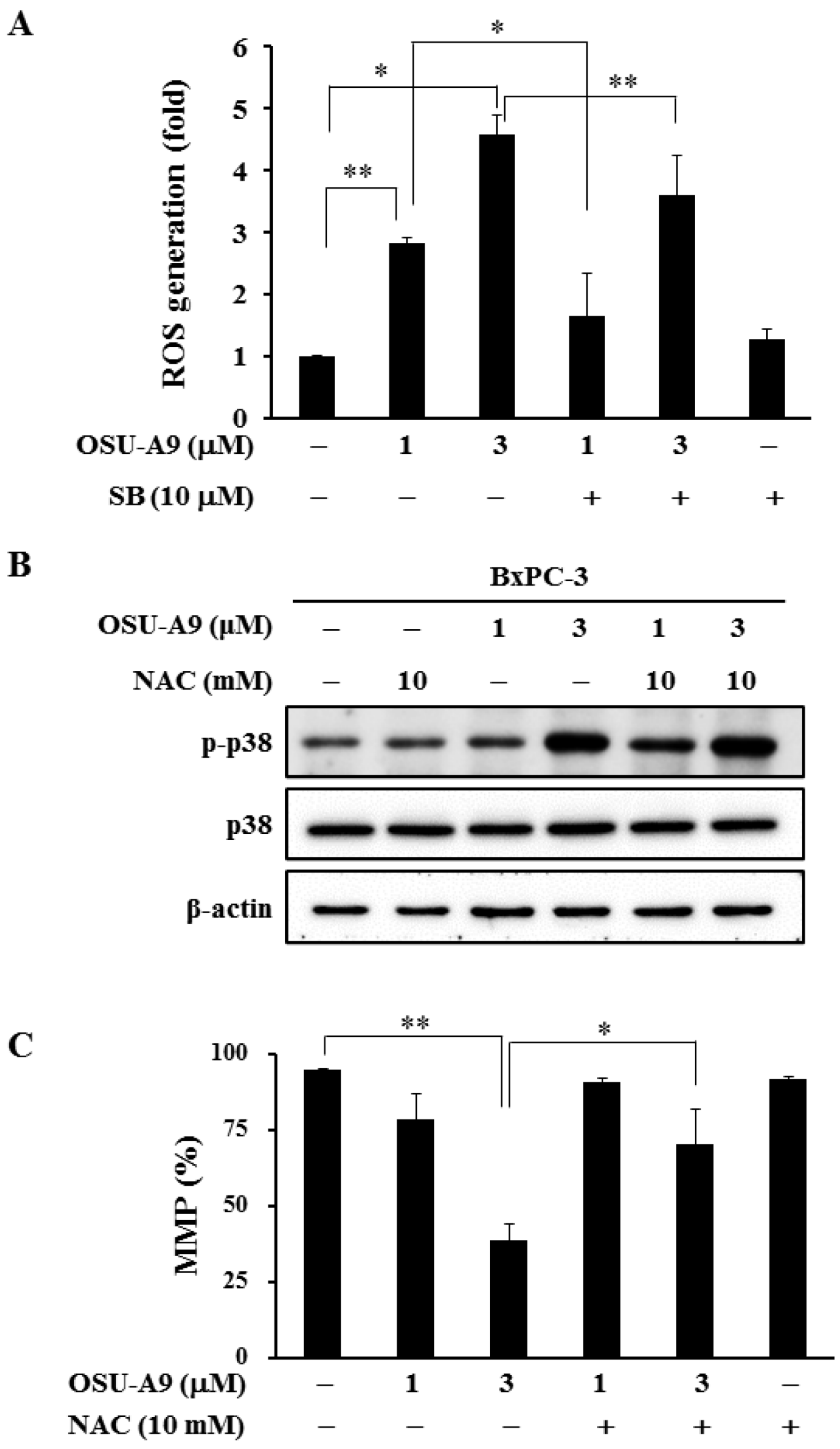

Supplementary Figure 2: Analysis of reactive oxygen species and mitochondrial membrane potential in BxPC-3 cells. **(A)** Cells were treated with DMSO, or OSU-A9 with or without SB203580 (SB) for 3 h, and stained with DCFH-DA followed by FACS analysis. *$P < 0.05$, ** $P < 0.01$. **(B)** *N*-acetylcysteine (NAC) on the phosphorylation and expression of p38 in OSU-A9-treated BxPC-3 cells. **(C)** Cells were treated with DMSO, or OSU-A9 with or without NAC for 3 h and stained with JC-1 followed by FACS analysis. *$P < 0.05$, ** $P < 0.01$.

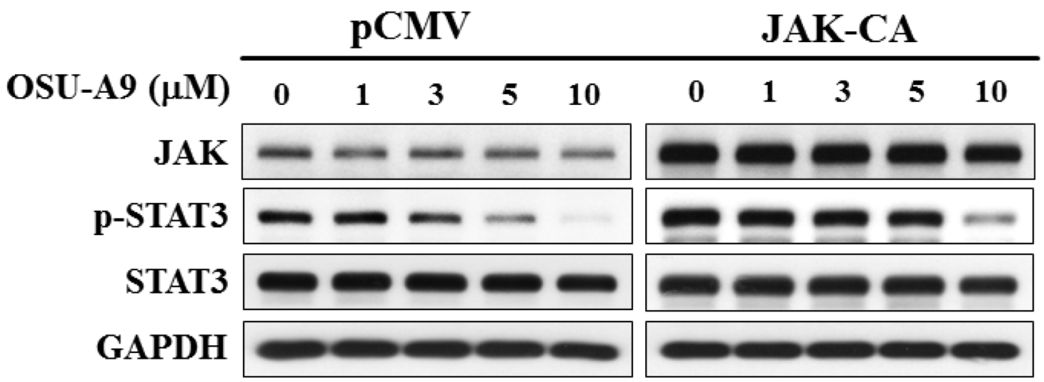

Supplementary Figure 3: Effects of the expression/phosphorylation of JAK and STAT3 in PANC-1 cells transfected with control vector (p-CMV) or constitutively active JAK (JAK-CA) and incubated with 0-10 μM OSU-A9 for 24 h.

Supplementary Table 1: ANOVA model for BxPC-3 cells treated with OSU-A9

OSU-A9	Degree of Freedom	Mean Square	F	P-value	Bonferroni Post-hoc tests (Between Dose condition)
Dose (μM)	3.42	53912.26	2039.88	<0.0001	2 < 0 (p<0.0001)<br>3 < 0 (p<0.0001)<br>5 < 0 (p<0.0001)<br>7.5< 0 (p<0.0001)<br>10 < 0 (p<0.0001)<br>2 < 0.5 (p<0.0001)<br>3 < 0.5 (p<0.0001)<br>5 < 0.5 (p<0.0001)<br>7.5< 0.5 (p<0.0001)<br>10 < 0.5 (p<0.0001)<br>2 < 1 (p<0.0001)<br>3 < 1 (p<0.0001)<br>5 < 1 (p<0.0001)<br>7.5 < 1 (p<0.0001)<br>10 < 1 (p<0.0001)<br>3 < 2 (p<0.0001)<br>5 < 2 (p<0.0001)<br>7.5 < 2 (p<0.0001)<br>10 < 2 (p<0.0001)<br>5 < 3 (p<0.0001)<br>7.5 < 3 (p<0.0001)<br>10 < 3 (p<0.0001)<br>7.5 < 5 (p<0.0001)<br>10 < 5 (p<0.0001)<br>10 < 7.5 (p<0.0001)
Time (hours)	2	2217.45	199.19	<0.0001	24 > 48 (p<0.0001)<br>24 > 72 (p<0.0001)
Dose*Time	6.83	680.16	25.74	<0.0001	-
Error	51.26	26.43	-	-	-

Supplementary Table 2: ANOVA model for PANC-1 cells treated with OSU-A9

OSU-A9	Degree of Freedom	Mean Square	F	P-value	Bonferroni Post-hoc tests (Between Dose condition)
Dose (µM)	2.98	43662.47	312.03	<0.0001	2 < 0 (p<0.0001)<br>3 < 0 (p<0.0001)<br>5 < 0 (p<0.0001)<br>7.5< 0 (p<0.0001)<br>10 < 0 (p<0.0001)<br>2 < 0.5 (p<0.0001)<br>3 < 0.5 (p<0.0001)<br>5 < 0.5 (p<0.0001)<br>7.5< 0.5 (p<0.0001)<br>10 < 0.5 (p<0.0001)<br>2 < 1 (p<0.0001)<br>3 < 1 (p<0.0001)<br>5 < 1 (p<0.0001)<br>7.5 < 1 (p<0.0001)<br>10 < 1 (p<0.0001)<br>3 < 2 (p<0.0001)<br>5 < 2 (p<0.0001)<br>7.5 < 2 (p<0.0001)<br>10 < 2 (p<0.0001)<br>5 < 3 (p<0.0001)<br>7.5 < 3 (p<0.0001)<br>10 < 3 (p<0.0001)<br>7.5 < 5 (p<0.0001)<br>10 < 5 (p<0.0001)<br>10 < 7.5 (p<0.0001)
Time (hours)	2	1134.99	5.87	0.013	24 > 72 (p=0.018)
Dose*Time	5.96	111.88	0.80	0.58	-
Error	44.68	139.93	-	-	-

Supplementary Table 3: Effects on hematological and serum biochemical parameters of 42 days administration of OSU-A9 in mice

	Vehicle	OSU-A9 (25 mg/kg)
**PCV (%)**	42.7±2.1	42.7±2.1
**Hemoglobin (gm/dl)**	13.9±0.7	14.1±0.7
**RBC (cells × 10^{12}/l)**	9.4±0.2	9.2±0.4
**MCV (fl)**	45.7±1.5	46±1
**MCHC (gm/dl)**	32.7±0.2	33±0.1
**RDW (%)**	17.8±1.9	17.7±0.4
**Seg. Neutrophils (cells × 10^{9}/l)**	43±20.2	42.3±13.4
**Lymphocytes (cells × 10^{9}/l)**	50.5±16.3	48.3±14.6
**Monocytes (cells × 10^{9}/l)**	11.7±3.8	6.7±3.2
**Eosinophils (cells × 10^{9}/l)**	0.7±1.2	3±2
**Platelet Count (10^{3}/µL)**	1554667±172247.9	1354336±120691.5
**BUN (mg/dl)**	20±0	18.7±4.2
**Creatinine (mg/dl)**	0.3±0.1	0.3±0.1
**Serum Osmolality (mOsm/L)**	320±2	322.7±6.4
**ALT (IU/l)**	30.7±2.3	44.7±16.2
**AST (IU/l)**	50.7±8.3	82±19.3
**CK (U/L)**	168.7±20	143.3±33.6
**Cholesterol (mg/dl)**	122±11.1	132.7±14.5
**Bilirubin, Total (mg/dL)**	0.13	0.14
**Total Protein (g/dl)**	5.7±0.2	6.1±0.2
**Albumin (g/dl)**	3.6±0.2	3.5±0.1
**Globulins (g/dl)**	2.1±0.2	2.6±0.2
**Glucose (mg/dl)**	238±7.2	178.7±26.6
